# The Minor Flagellin of *Campylobacter jejuni* (FlaB) Confers Defensive Properties against Bacteriophage Infection

**DOI:** 10.3389/fmicb.2016.01908

**Published:** 2016-11-29

**Authors:** Lukas Lis, Ian F. Connerton

**Affiliations:** ^1^PTC Phage Technology Center GmbH, Im Kompetenzzentrum BioSecurityBönen, Germany; ^2^Division of Food Sciences, School of Biosciences, University of NottinghamLoughborough, UK

**Keywords:** *Campylobacter*, bacteriophage, flagellin, flaB, phage escape

## Abstract

A screen of bacteriophages infecting a panel of *Campylobacter jejuni* PT14 gene knock-out mutants identified a role for the minor flagellin encoded by the *flaB* gene, in the defense of the host against CP8unalikevirus bacteriophage CP_F1 infection. Inactivation of the *flaB* gene resulted in an increase in the susceptibility of PT14 cultures to infection by CP_F1 and an increase in bacteriophage yields. Infection of wild type PT14 with CP_F1 produces turbid plaques in bacterial lawns, from which 78% of the resistant isolates recovered exhibit either attenuation or complete loss of motility. CP_F1 produces clear plaques on the *flaB* mutant with no regrowth in the lysis zones. Complementation of the mutant restored overgrowth and the development of resistance at the expense of motility. Further analyses revealed an increase in bacteriophage adsorption constant of nearly 2-fold and burst-size 3-fold, relative to the wild type. Motility analysis showed no major reduction in swarming motility in the *flaB* mutant. Thus, we propose a new role for FlaB in the defense of campylobacters against bacteriophage infection.

## Introduction

*Campylobacter* represents a major zoonotic pathogen, as emphasized in recent reports published by the European Food Safety Authority that document year-on-year recorded caseloads of >210.000, which are estimated to belie an actual annual infection rate of 9 million people (EFSA, 2012, 2014, 2015). Human infection by this gram-negative bacterium leads to gastroenteritis (termed campylobacteriosis) with symptoms including severe abdominal pain, fever and diarrhea (Blaser, 1997; Allos, 2001). The infection is generally self-limiting and can be treated with rehydration therapy. However, in rare cases chronic sequelae can develop, for example reactive arthritis or Guillain-Barré syndrome (GBS), autoimmune disorders that can lead to temporary or in severe cases permanent paralysis (Nachamkin, 2002). The fact that campylobacters are highly prevalent in the intestinal tracts of farm livestock, such as poultry and pigs, renders it a major foodborne pathogen, and therefore represents a significant risk to consumer health (Boes et al., 2005; McCrea et al., 2005).

In order to effectively colonize their host, *Campylobacter* cells are equipped with one or two polar located flagella that confer high motility. Recent estimates suggest a torque of 3600 pN/nm for *C. jejuni* flagella, which is more than twice as high as that reported for *Salmonella* cells (Beeby et al., 2016). These high mobility structures together with CadF and FlpA adhesins help withstand peristaltic forces in the intestines of its colonized host, preventing them from being expelled and to facilitate locomotion in the highly viscous environment of the epithelial cell mucus layer (Monteville et al., 2003; Konkel et al., 2004, 2010). The invasion of intestinal epithelial cells is another key function of the flagella apparatus. A model proposed for *Campylobacter* pathogenesis suggests attachment to and disruption of epithelial cell barriers, before migrating toward the basal ends of cells, where incorporation takes place (O'Loughlin and Konkel, 2014). For this purpose, the flagella act as a type III secretion apparatus releasing Cia proteins (*Campylobacter* invasion antigens), that induce the cell invasion process (Konkel et al., 2001). Through these functions flagella are recognized as crucial for colonization and virulence (Bolton, 2015).

Interestingly, these motility structures have also been employed by the natural antagonists of *Campylobacter*, as they serve as attachment sites for bacteriophage infection. It has been shown that loss of the flagellin structure or motility function will result in resistance to infection from flagellotropic bacteriophages (Coward et al., 2006; Scott et al., 2007; Baldvinsson et al., 2014). Javed et al. (2015) further supported these findings by reporting that glycosylated flagellin serves as target for a phage receptor-binding protein (RBP) from bacteriophage NCTC12673. The main initial binding-target of flagellotropic phages, namely the rotating flagellar filament, is coded for by two tandem genes *flaA* and *flaB*, which show 95% sequence identity (Wassenaar et al., 1991). Selective disruption of the major flagellin gene (*flaA*) or a double deletion mutant lead to loss of the full-length flagellar filament, which results in the loss of motility, reduced colonization efficiency and resistance to flagellotropic bacteriophages (Guerry et al., 1991; Nachamkin et al., 1993; Baldvinsson et al., 2014). Inactivation of *flaB* does not have a major impact on motility as the FlaB content within the flagellar filament is sparse (Alm et al., 1993). Expression of the alternative flagellin genes are governed by different sigma factors. While *flaA* is under control of σ^28^, *flaB* is σ^54^ regulated (Nuijten et al., 1990). The functional significance or benefits of this separate gene regulation have yet to be unraveled. There are suggestions that *flaB* is involved in an intragenomic recombination mechanism aiming to evade immunological responses of the colonized host, and therefore increasing antigenic diversity (Harrington et al., 1997). Whether this could also have an effect on bacteriophage infection has not been tested, since there are no data available in literature concerning links of the minor flagellin gene product (FlaB) to bacteriophage infection.

The aim of the present study was to identify factors that impact on Eucampyvirinae bacteriophage infection that constitute a virulent subfamily of the *Myoviridae* (Javed et al., 2014), and are candidates for phage therapy of farm animals (Loc Carrillo et al., 2005; El-Shibiny et al., 2009; Kittler et al., 2013; Hammerl et al., 2014) and phage biosanitization applications (Atterbury et al., 2003; Goode et al., 2003; Bigwood et al., 2008) to control campylobacters in the human food chain (Connerton et al., 2011). For this purpose, we have constructed isogenic knock-out mutants targeting virulence and animal host colonization factors in the bacteriophage propagation strain *C. jejuni* PT14 (Brathwaite et al., 2013). We report for the first time an effect of the *flaB* gene product in connection to bacteriophage infection. In the absence of functional FlaB we observed an increase in susceptibility to infection by bacteriophage CP_F1 and increased phage propagation after a 24 h incubation period in liquid culture. Further, we observed changes in the adsorption rate and burst size that suggests FlaB has a defensive role against bacteriophage infection.

## Materials and methods

### Bacteriophage, bacterial strains, and growth conditions

Bacteriophage CP_F1 was originally isolated from a pig manure sample and was propagated on *C. jejuni* PT14 (Brathwaite et al., 2013). All strains, associated plasmids and oligonucleotide PCR primers used in this study are listed in Table 1. The *C. jejuni* strains were routinely grown on blood agar base No. 2 plates (BA plates; Oxoid) supplemented with 5% horse blood (TCS Biosciences) under microaerobic conditions in either a modular atmosphere controlled cabinet (5% CO_2_, 5% O_2_, 2% H_2_, 88% N_2_) or in anaerobic jars using gas replacement (7.3% CO_2_, 5.6% O_2_, 3.6% H_2_, 83.5% N_2_) at 42°C. *Escherichia coli* strain TOP10 (Invitrogen) was used for cloning and cultured aerobically at 37°C on Luria agar plates or in Luria broth containing appropriate antibiotic as required.

**Table 1:** **Bacterial strains, plasmids, and primers used in this study**.

**Strain**	**Characteristic or genotype**	**Sources**
*C. jejuni* NCTC12662 PT14	propagating strain for bacteriophages CP_F1, CP220	National Collection of Type Cultures
*C. jejuni* NCTC12662 PT14 Δ*flaA*	*ΔflaA::Kan*	This study
*C. jejuni* NCTC12662 PT14 Δ*flaAB*	*ΔflaAB::Kan*	This study
*C. jejuni* NCTC12662 PT14 *kpsM*	*kpsM::Kan*	This study
*C. jejuni* NCTC12662 PT14 *flaB*	*flaB::Kan*	This study
*C. jejuni* NCTC12662 PT14 *flaB, 0046::flaB*	*flaB::Kan, 00230::flaB cat*	This study
*C. jejuni* NCTC11168H *flaA*	*ΔflaA::Kan*	Jones et al., 2004
*C. jejuni* NCTC11168 *flaAB*	*ΔflaAB::Kan*	M. Jones Univ of Nottingham
*C. jejuni* NCTC11168 *flaB*	*flaB::Kan*	M. Jones Univ of Nottingham
*C. jejuni* NCTC11168 *kpsM*	*kpsM::Kan*	Karlyshev et al., 2000
*E. coli* TOP10	Plasmid propagation strain	Invitrogen
**PLASMIDS**	**CHARACTERISTIC**	
pCfdxA	*cj0046*::*cat*	Gaskin et al., 2007
pC_flaB	*cj0046*::*flaB cat*	This study
**PRIMERS**	**NUCLEOTIDE SEQUENCE**	
flaA_fw	GATTGCACGATATAGCATTTAACAAG	
flaA_rv	TCTAAACTAGGCTTGGATTTGTA	
flaA_int_fw	AGGACTTGGAGCTTTAGCAGATGAGAT	
flaA_int_rv	CGGCAGTATTAGCATCAAGCTGTC	
flaA_i5′_rv	GTAAGATACCTAAAGCATCGTTAC	
flaB_fw	GCCATGGCACAGGCTAATTC	
flaB_rv	GGGTTTATGCACACGAAGCTTTGATAG	
flaB_int_fw	AACGACCATAATTTTCCATCATATTTG	
flaB_int_rv	GGTGAAGTGCAATTTACTCTTAAAAATTAC	
kpsM_fw	AAGGTGTGCAAGCTAAGGCCGAGTT	
kpsT_rv	GATCTCCAACAGCTCCTGCTTCAT	
flaB_NgoMIV_fw	CGGCCGGCACAAATCCAAGCCTAGTTTAGAA^*^	
flaB_BsmBI_rv	AACGTCTCACATGGACGAAGCTTTGATAGAAAATATCAT^*^	
cj0046_fw	GCTCTTAGTGGCATTACCACTACC	
cj0046_rv	GCCACACTAGTCGCATCAAGAGAA	

* *Restriction sites are underlined*.

### Construction of *C. jejuni* PT14 mutant and complemented strain

#### Transformation of *C. jejuni*

Site-specific knock-out mutations in *C. jejuni* PT14 were generated by transformation with genomic DNA from established and verified constructs in donor strains (Table 1). As most strains of *C. jejuni* exhibit efficient natural transformation, the protocol from Wang and Taylor (1990) was employed. The *C. jejuni* PT14 overnight cultures were collected from BA plates and dissolved in Mueller Hinton (MH) broth (Carl Roth). Cells were adjusted to OD_600_ 0.5 (corresponding to approx. 3 × 10^9^ CFU/ml) and aliquots of 500 μl of adjusted culture were added to 1 ml of MH broth in a 15 ml tube. Following incubation at 42°C under microaerobic conditions for 5 h, 1–5 μg of extracted chromosomal DNA (GenElute Bacterial Genomic DNA Kit, Sigma Aldrich) was added and cells were further incubated overnight. On the following day, cells were concentrated by centrifugation at 13,000 × *g* for 15 min. Pellets were resuspended in 100 μl of MH broth and spread on BA plates containing the selective agent according to the selective marker in use (34 μg/ml chloramphenicol or 50 μg/ml kanamycin from Sigma Aldrich). Selection plates were then incubated for 24–48 h at 42°C under microaerobic conditions. Genomic insertion of the desired mutant genes were verified via PCR amplification and DNA sequencing.

#### Trans complementation of the *flaB* mutant strain

To achieve a trans-complementation of the disrupted *flaB* gene in strain *C*. *jejuni* PT14, a suicide vector based on pCfdxA (Gaskin et al., 2007) was constructed. For this purpose, the wild type *flaB* gene and the region 100 bp upstream carrying the native promoter were PCR amplified from the genomic DNA of *C. jejuni* PT14 with primers flab_NgoMIV_fw and flab_BsmBI_rv. After restriction digest the fragment was cloned into the vector adjacent to a chloramphenicol resistance cassette (*cat*). This vector further harbored regions of pseudogene *cj0046* from *C. jejuni* NCTC11168 flanking the cassette and the gene of interest which enables recombination with the homologous pseudogene A911_00230 of strain *C*. *jejuni* PT14. Complementation of the mutant strain *C. jejuni* PT14 *flaB::kan* was performed by electroporation, as natural transformation of *E. coli* derived plasmids was not efficient. Colonies were sub-cultured and analyzed for positive integration by colony PCR using primers cj0046_fw and cj0046_rv that bind in the flanking regions of the A911_00230 pseudogene. Additionally, the amplicon was gel purified and sequenced for additional verification.

### Spot test assays and titer determination

A determination of bacteriophage infection of the *C. jejuni* PT14 wild type and mutant strains was performed by employing the traditional spot test assay. A fresh overnight culture of the test *Campylobacter* strain was collected from a BA plate and emulsified in MH broth supplemented with 10 mM MgSO_4_ (Thermo Fisher) to a cell density of approximately 10^7^ CFU/ml. A volume of 500 μl of emulsified cells was added to 5 ml of molten NZCYM (0.6%, Carl Roth) top agar and poured on top of a NZCYM agar plate. After the top agar had solidified, 5 μl aliquots of the phage suspensions, at the routine test dilution of approximately titer of 10^7^ PFU/ml (Frost et al., 1999), were dispensed on top of the soft agar layer. The plate was then incubated at 42°C under microaerobic conditions for 24–48 h after the spots dried into the agar. In a similar way the phage titer was determined by serial 10-fold dilutions of phage suspensions, which were applied as 10 μl droplets in triplicate to the surface of prepared host bacterial lawns and allowed to dry. After incubation the plaques were counted and titers calculated.

### Motility assays

In order to assess swarming motility, fresh overnight cultures were taken from a BA plate and resuspended in MH broth. After adjustment of cell suspensions to OD_600_ 0.1, 2 μl of suspension was removed with a pipette tip and stabbed into a swarming agar plate (0.4% Mueller Hinton agar). Inoculated plates were then incubated under microaerobic conditions at 42°C for 48 h. Growth zones were measured for each of triplicate samples after 24 and 48 h to assess cell motility.

### Analysis of bacteriophage infection in liquid growth media

The growth from *C. jejuni* BA plates incubated overnight at 42°C were collected and emulsified in PBS buffer (Merck Millipore) for the adjustment of inoculum concentrations. Flasks containing 50 ml nutrient broth No. 2 (Oxoid) were inoculated with *C. jejuni* cells at starting concentrations of approximately 10^5^ or 10^7^ CFU/ml. After inoculation, flasks were sealed with cotton stoppers and placed in anaerobic jars (Oxoid). Microaerobic conditions were introduced using the gas replacement method (John et al., 2011). Cultures were then incubated in a shaking incubator at 42°C and 100 rpm. After 2 h incubation, bacteriophage CP_F1 was added at an MOI of 2. Samples were collected at 2 h intervals for determination of viable cell counts and phage titers using the Miles and Misra method. Phage samples were treated with 2 μl chloroform (Thermofisher) and centrifuged at 13,000 × *g* for 5 min. The supernatant was removed and used for titration. The *C. jejuni* PT14 wild type and knock-out strains were tested in triplicate to ensure statistical certainty. Growth controls without phage addition served as references.

### Adsorption assay

Phage adsorption rates were determined as described previously (Siringan et al., 2014) with minor modifications outlined as followed. Suspensions of *C. jejuni* PT14 wild type, *flaB* mutant and trans-complemented *flaB* cells that contained approximately 5 × 10^7^ CFU/ml, were inoculated in nutrient broth No. 2 and incubated at 42°C under microaerobic conditions at 100 rpm for 5 h to obtain cells in the exponential growth state. The actual viable count was determined following serial dilution and incubation as described above. For the experiment, bacteriophage CP_F1 was diluted and added to the suspensions to give a final titer of 10^5^ PFU/ml, then briefly mixed and kept static at 42°C under microaerobic conditions. Sampling was performed every 5 min over a period of 30 min. Samples were immediately centrifuged at 13 000 × *g* for 5 min and the supernatants removed. The titer of free bacteriophages in the supernatants was determined and used to calculate numbers of bound bacteriophages. Bacteriophage adsorption constants were determined using the formula *k* = -ln (P_*t*_/P_0_)/Nt, where *P*_*t*_ = phage titer at time point t (PFU/ml), *P*_0_ = initial phage titer (PFU/ml), *N* = bacterial density (CFU/ml) and *t* = time (min).

### Efficiency of plating of phage CP_F1 on mutant strains

The efficiency of plating (EOP) of bacteriophage CP_F1 infecting the PT14 mutant strains was determined by enumerating bacteriophages as described above. Subsequently the calculations were made by dividing the bacteriophage titer obtained when applied to the lawns prepared from different mutant strains by the titer obtained when applied to *C. jejuni* PT14 wild type lawns.

### Bacteriophage growth parameter determination

In order to assess the burst size and latent period for bacteriophage CP_F1 infection of *C. jejuni* PT14 wild type, the *flaB* mutant and trans-complemented derivatives, single step growth curves were monitored over 4 h. The protocol after Carvalho et al. (2010) was employed for this purpose. Triplicate samples of 10 ml nutrient broth No. 2 were inoculated with 10^7^ CFU/ml cells and grown under microaerobic conditions at 42°C under constant shaking at 100 rpm into early exponential phase (approx. 10^8^ CFU/ml). Bacteriophage CP_F1 was added at an MOI of 0.001. Samples for titer determination were taken every 15 min for 4 h. These were centrifuged at 13,000 × *g* for 5 min and the supernatants used for titration. Three replicates of each individual experiment were performed and mean values used for presentation of titer development. Non-linear regression was used to determine latent period and burst size of the first burst event.

### Whole genome sequencing

Genomic DNAs from *C. jejuni* PT14 non-motile variants were prepared using the GenElute Bacterial Genomic DNA Kit (Sigma Aldrich) following the manufacturer's instructions. DNA sequencing was performed using the Illumina MiSeq platform. The data consisted of 3.1–4.4 million paired-end sequence reads of 250 bp in length. Initial processing of the raw data, mapping of the sequence reads to *C. jejuni* PT14 (GenBank accession number CP003871) and variant detection were performed using CLC Genomics Workbench version 8.0 (Qiagen, Aarhus, Denmark).

### RNA extraction

Total RNAs were extracted from CP_F1 infected and uninfected control cultures of *C. jejuni* PT14. For each treatment, three independent early-log phase cultures (eclipse phase of the infected cultures) growing in nutrient broth No. 2 were harvested 50 min after phage addition and the RNA content extracted using TRIzol Max with Max Bacterial Enhancement Reagent (Invitrogen) according to the manufacturer's protocol. Subsequently ethanol-precipitation and purification using the RNeasy Mini kit (Qiagen, Crawley, UK) according to the manufacturer's instructions were performed including DNase treatment using RNase-free DNase and related reagents (Qiagen). Pure RNA samples were collected in 40 μl of RNase free water and analyzed for quantity/purity (Nanodrop ND1000) and quality (Bioanalyser 2100, Agilent Technologies Inc.). Prokaryote Total RNA Nano series II software, Version 2.3 was used for analysis of the RNA quality. All purified RNA samples showed a RNA Integrity Number (RIN) of 10.0.

### Real time qRT-PCR

Total RNAs were converted to cDNA with random hexamer primers using the Superscript II (Fisher Scientific) reverse transcriptase system according to the manufacturer's protocol. Specific primers for qRT PCR were designed with lengths of 18–24 nt and can be found in Supplementary Table S1. An optical 48 well microtiter plate (Applied Biosystems) was used with 20 μl reaction volumes consisting of Power SYBR Green PCR master mix (Life Technologies), 50 nM gene specific primers and 100 ng of the cDNA template. A StepOne real time PCR system (Applied Biosystems) was programmed for an initial set up of 30 s at 95°C, followed by 40 cycles of 15 s at 95°C and 1 min at 58°C. A melting curve was obtained from 50 to 95°C to control specificities of quantitative PCR reaction for each primer pair. Cycle threshold (CT) values were determined employing the StepOne software version 2.0 (Applied Biosystems). The comparative threshold cycle method was used to calculate change (n-fold) where samples were normalized to the internal control product of the 50 S ribosomal subunit protein L1 gene (*rplA*), which showed no change in expression levels between phage infected and uninfected cultures of *C*. *jejuni* PT14 during acute CP8 infection in previous experiments. Reactions were performed in triplicate. The fold changes were calculated using the 2^−ΔΔCt^ method. Verification of acute phage infection was confirmed by PCR using synthesized cDNA, employing specific primers targeting the gene for the major capsid protein (mcp) of bacteriophage CP_F1 (homolog of gp23 from phage NCTC12673; Kropinski et al., 2011).

### Statistical treatment of data

Statistical differences between paired control and treatment groups were assessed by using the Student's *t*-test with significance *p* < 0.05. Differences between experimental groups were analyzed by analysis of variance. Viable count and phage titer data were log_10_-transformed for analysis.

## Results

### Disruption of *flaB* yields clear lysis zones for bacteriophage CP_F1

**Figure 1:**
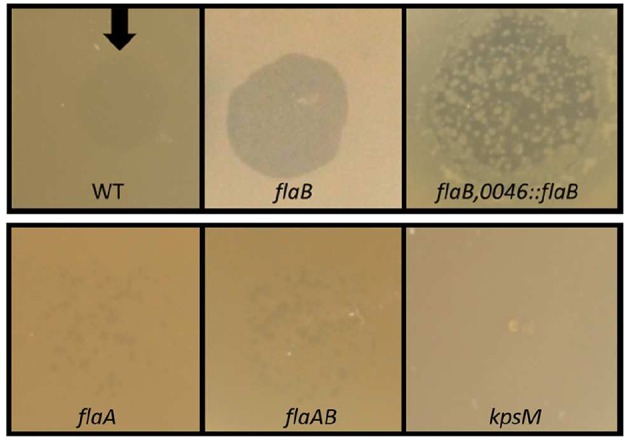
**Lysis zones of bacteriophage CP_F1 on bacterial lawns of ***C. jejuni*** PT14**. Opaque lysis areas were observed upon infection of the wild type strain caused by regrowth of a *Campylobacter* sub-population less susceptible to phage infection. Disruption of the *flaB* gene resulted in clear lysis. Complementation of the *flaB* mutant partially restored opaque lysis. Reduced plating efficiency and opaque plaques were observed in *flaA* and *flaAB* mutants. No plaque formation occurred for the *kpsM* mutant.

By screening bacteriophage infection of a number of mutant variants of *C. jejuni* PT14 we have analyzed the effects of flagella related factors on this process. Spot test assays showed a clear dependence on rotating flagella for bacteriophage CP220 (prototype of the Cp220likevirus genus), as no replication and host lysis was observed for *flaA* or *flaAB* mutants (Supplementary Figure S1). Similarly, the CP8unalikevirus phage CP_F1 showed decreased infection efficiency on non-motile cells, as infection of *flaA* and *flaAB* mutant strains yielded reduced efficiency of plating (EOP) values (*flaA* 0.08 ± 0.04, *n* = 3; *flaAB* 0.12 ± 0.03, *n* = 3) and highly opaque plaques. Additionally we observed CP_F1 to be strictly dependent on capsular polysaccharide (CPS) as no plaque formation occurred on a *kpsM* mutant (Figure 1). Interestingly, we found an aberration in infection by bacteriophage CP_F1 of a *flaB* disruption mutant. CP_F1 was isolated from a pig sample and propagated on strain PT14. It is able to infect and propagate on its host, but yields opaque plaques on soft agar bacterial lawns, which reveal regrowth of the host after infection. We observed similar reactions for phage CP220 (Supplementary Figure S1), suggesting that this reaction is not limited to one class of *Campylobacter* phage (Javed et al., 2014). However, propagation on a *flaB* mutant of PT14 produced clear lysis zones that persisted even after prolonged incubation for 24 h (Figure 1). Five individual clones, which all carried the desired disruption of *flaB* were tested in spot test assays to exclude effects of secondary mutations (de Vries et al., 2015). All showed identical phenotypes toward phage-induced lysis on soft agar lawns and motility (Supplementary Figure S2).

**Figure 2:**
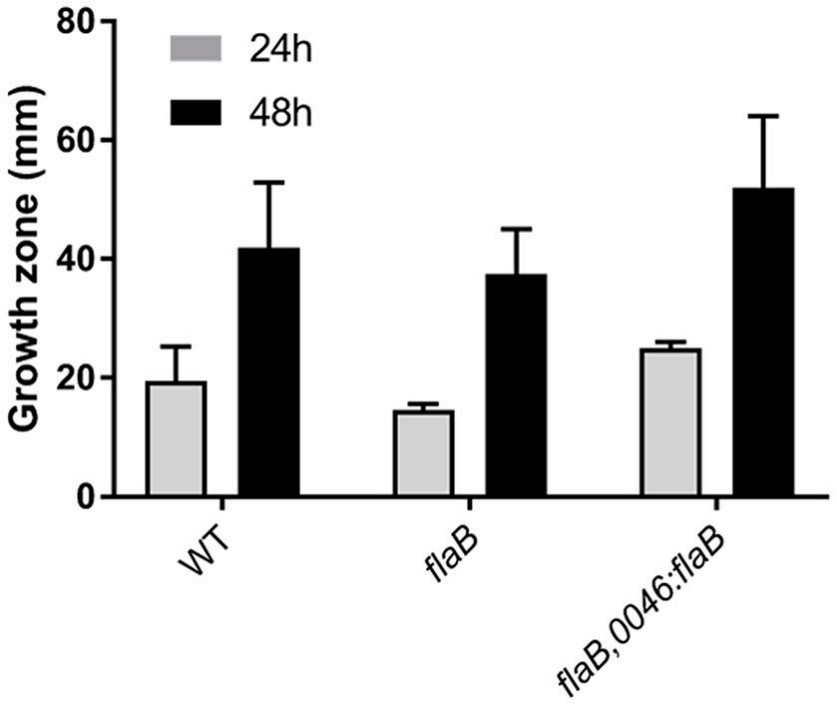
**Swarming motility of ***C. jejuni*** PT14 wild type (WT), ***flaB*** mutant and the complemented strain on low density agar**. The diameters of growth zones after 24 and 48 h of incubation are derived from 3 biological replicates and presented as means with standard deviations.

DNA sequencing of PCR amplicons of all five clones carrying the desired gene disruption, revealed an integration of the resistance marker in the *flaB* gene in the orientation of the *flaB* reading frame, mitigating any polar effects on the adjacent *flaA* gene. We also detected no mutations in the *flaA* gene, as further supported by findings from motility tests. Analysis of the motility of the *flaB* mutant strains revealed no major reduction in swarming motility (Figure 2), which is in accordance with previous observations (Wassenaar et al., 1991; de Vries et al., 2015).

To analyze the regrowth of *Campylobacter* cells in lysis zones we collected and sub-cultured surviving cells from the surface of turbid spots. Single colony isolates of these were examined for motility, reaction to phage infection by CP_F1 and DNA sequence analysis of the *flaA/B* region. We found that the majority of clones (78%, *n* = 100) showed either complete loss of motility or attenuation of their swarming ability (Supplementary Table S2), which was accompanied by resistance to reinfection by phage CP_F1. Additionally, eight clones were observed to have shifted into the carrier state (Siringan et al., 2014), as demonstrated by plaque formation inside of the growth zone on the motility agar plates and detectable phage propagation after serial sub-culturing of these clones (Supplementary Figure S3). PCR amplification and DNA sequence analysis of the *flaA* gene of the non-motile isolates revealed no alteration in the gene or non-coding sequences. The non-motile isolates were serially sub-cultured and after each passage the motility and phage resistance of the culture were assessed by inoculation of swarming motility agar plates and plaque assay. After two rounds it was observed that cultures arising from a single clone had recovered motility. In one case the process of escape from bacteriophage infection was reversible, which could be attributable to phase variation. Whole genome sequencing of three non-motile clones exhibiting phage resistance revealed all to have suffered a single nucleotide deletion in the N-terminal region of the *rpoN* gene coding for the RNA polymerase factor σ^54^ (A911_03265) at nucleotide position 244–250 in a stretch of 7 adenine residues. Further several phase variable genes were shifted to the off state compared to the wild type genome sequences (Table 2). For the *flaB* mutant, the shift to a phage resistant population could not be observed, which we assume is due to an increased susceptibility of the mutant to phage infection that may also be accompanied by an inability to support the mutation(s) leading to phage resistance.

**Table 2:** **Observed single nucleotide changes in the genome sequences of escape mutants recovered after CP_F1 infection relative to the reference sequence of ***C. jejuni*** PT14 (CP003871)**.

**Accession number**	**Gene product**	**Changes in coding region**	**Phase status**	**Occurance in clones**
A911_03265	RNA polymerase factor sigma-54	A deletion pos.244	–	1,2,3
A911_03335	invasion protein CipA	C insertion pos.844_845	phase off (10C)	2,3
A911_05520	1,3-galactosyltransferase	G deletion pos.341	phase off (10G)	1,2,3
A911_06290	Hypothetical protein	G insertion pos.167_168	phase off (10G)	1,2,3
A911_06295	Aminoglycosidase N3′-acetyltransferase	G deletion pos.310	phase off (9G)	2
A911_06340	Hypothetical protein	G insertion / deletion pos.588	phase off (12C/10C)	2 (ins), 3 (del)
A911_06345	Hypothetical protein	G insertion pos.588_589	phase off (10C)	1,2,3
A911_06440	put. methyl transferase	G insertion pos.251_252	phase off (10G)	1,2,3
A911_06490	Motility accessory factor	G insertion pos.168_169	phase off (10G)	1,2,3
A911_06906	SAM dependent methyltransferase	G deletion pos.402	phase off (8C)	1,2,3
A911_06907	put. sugar transferase	G insertion pos.123_124	phase on (8C)	1,2,3
A911_07000	alpha-2,3-sialyltransferase	G insertion pos.1605_1606	phase on (10G)	1,3
A911_08080	Lipoprotein	G deletion pos.497	phase off (9G)	1,2,3

*Nine out of thirteen coding regions feature identical nucleotide changes in all three clones*.

### Complementation leads to partial restoration of the wild type phenotype

In order to verify that the observed increase in susceptibility toward phage infection was a result of the disruption of the *flaB* gene, we constructed a plasmid vector for the introduction of an intact copy of *flaB* into the pseudogene A911_00230 of the mutant strain. This construct was used to transform the *flaB* mutant strain and the correct insertion verified by DNA sequencing the target region. Trans-complementation of the disrupted *flaB* gene led to partial restoration of the phenotype with respect to bacteriophage infection. Overgrowth was restored within phage lysis zones but was less turbid than the wild type. The swarming motility of the complemented strain remained comparable to wild type but the survivors of phage infection were compromised in motility (Figure 2).

### Increased susceptibility and burst size in liquid growth cultures

**Figure 3:**
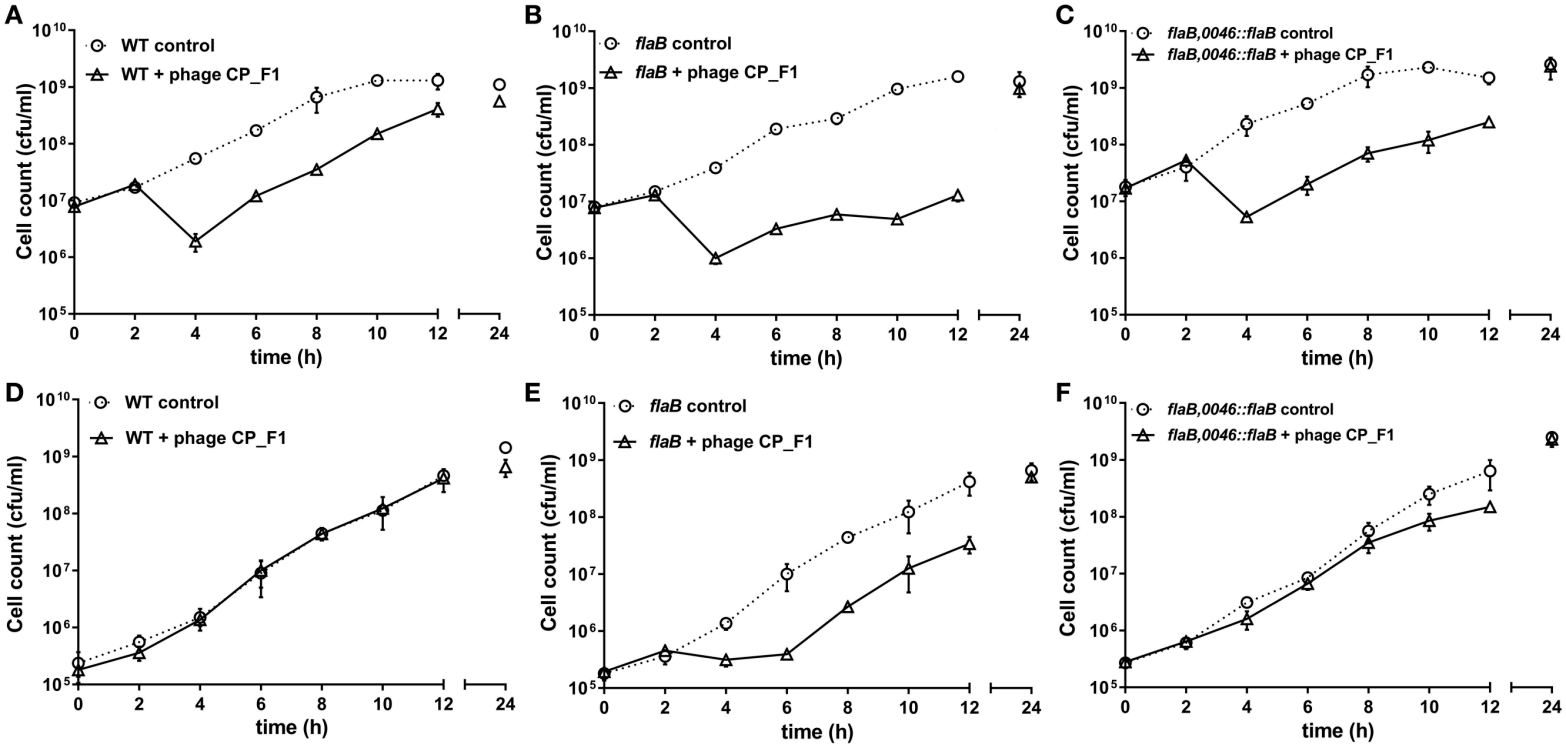
**Growth profiles of ***C. jejuni*** PT14 wild type strain (A,D)**, flagellin B mutant **(B,E)** and trans-complemented *flaB* cultures **(C,F)** in nutrient broth No. 2. Phage infection was accomplished by addition of bacteriophage CP_F1 to achieve an MOI 2 whilst uninfected cultures were mock infected. Panels **(A–C)** show initial infection above the phage proliferation threshold and panels **(D–F)** below the phage proliferation threshold. Data points indicate mean values for *n* = 3 biological replicates and the error bars show the standard deviation.

In order to analyze the changes in phage susceptibility in greater detail, phage replication experiments were performed in 50 ml liquid medium cultures, using either wild type or the *flaB* mutant or the *flaB* trans-complemented strain of *C. jejuni* PT14 over a period of 24 h (Figure 3). We tested the effects on phage infection at two different host cell densities, above and below the phage proliferation threshold of log_10_ 7 CFU/ml, which represents the density of bacteria required for the productive replication of bacteriophage (Cairns et al., 2009). At higher cell densities CP_F1 infection of wild type, *flaB* mutant and *flaB* complement cultures resulted in an initial fall of approximately 1 log_10_ CFU/ml in the viable count. This event was followed by a recovery in the viable count of wild type cultures (Figure 3A). In contrast the *flaB* mutant exhibited a drastic reduction in the growth rate (μ) post the phage-induced crash in viable count (Figure 3B). The growth rates post population crash for infected wild type μ = 0.67 ± 0.02/h and infected *flaB* mutant μ = 0.31 ± 0.05/h cultures (*p* < 0.01). The *flaB* complement culture also recovered faster than the mutant (Figure 3C) exhibiting a significantly greater growth rate of μ = 0.44 ± 0.06/h (*p* < 0.05). Phage proliferation at high initial cell densities showed significant differences between wild type and *flaB* phage-infected cultures with significantly greater phage yields at 24 h (*p* < 0.01).

**Figure 4:**
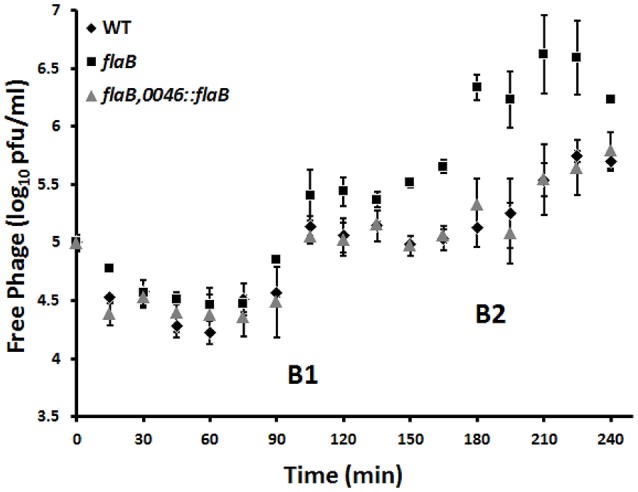
**Determination of burst size of ***Campylobacter*** bacteriophage CP_F1 infecting ***C. jejuni*** PT14 wild type (WT), flagellin B mutant (***flaB***) and the ***flaB*** trans-complement (***flaB, 0046::flaB***)**. Cultures were infected with a MOI of 0.001. Two notable rises in phage titer are marked as burst steps B1 and B2. Calculations of burst size, during the first burst event (90–105 min), resulted in a mean value of 1.4 virions (±0.4) per cell for the wild type strain, 1.8 virions (±0.6) per cell for the *flaB* trans-complement and 4.2 virions (±1.2) for the flagellin B mutant. Data points represent mean values for *n* = 3 independent biological replicates. Error bars represent standard deviations.

Infection of wild type *C. jejuni* PT14 at a bacterial density below the phage proliferation threshold with CP_F1 at an MOI of 2 resulted in minimal differences in the viable bacterial counts between infected and control cultures (Figure 3D). In contrast, the viable count for *flaB* mutant cultures remained static for a period of 4 h, post phage infection but subsequently these cultures entered exponential growth (Figure 3E). The growth rates of the infected cultures post delay were not significantly different to the uninfected control cultures (control: μ = 0.62 ± 0.10/h; infected: μ = 0.74 ± 0.06/h) suggesting under these conditions there was no fitness disadvantage for the emergent phage resistant sub-population. The delay evident in the phage infected *flaB* mutant was not present in trans-complemented cultures, which behaved similar to wild type with similar growth rates to the uninfected control at early stages of growth (Figure 3F). Analysis of phage propagation showed no increase in phage numbers during the first 12 h of incubation. However, after 24 h an increase in free virions was detected, implying phage replication. There was a significant difference in the phage titer of approximately 1 log_10_ PFU/ml (*p* < 0.001) between infected wild type cultures and the infected flagellin B mutant strain after 24 h (Supplementary Figure S4). Further analyses of the phage propagation parameters were examined using a one-step growth experiment. The latent period for CP_F1 was determined as 90 min with two burst events, marked B1 (90–105 min) and B2 (180–210 min) in Figure 4, evident over the 240 min period. The latent period for the CP_F1 infection process was in a comparable range to other studies (Cairns et al., 2009; Carvalho et al., 2010). The number of phage particles liberated per cell showed an increase for CP_F1 infecting the *flaB* mutant strain relative to the wild type and *flaB* complement. A burst size of 1.4 ± 0.4 PFU/cell in the wild type and 4.2 ± 1.2 PFU/cell in the mutant strain were calculated for the first burst event. Differences in the phage yields between the phage infected cultures were statistically significant (*p* < 0.05). Further, the phage yields were magnified after a second burst event. This resulted in a difference of 1 log_10_ in PFU between mutant culture and the wild type or the *flaB* complement at 225 min after the initial addition of the phage. However, the significant change in the replication process was not accompanied by a major difference in the relative efficiency of plating of phage CP_F1 between infection of the PT14 wild type and the flagellin B mutant strain (1.59 ± 0.38, *n* = 3).

To test if any changes in flagellin gene expression were induced as a response to acute phage infection we performed quantitative RT-PCR analyses. No alterations in the transcript levels of *flaA* or *flaB* in wild type PT14 were observed during the eclipse period of phage infection in which phage DNA replication and capsid assembly occurs prior to host lysis. A time point of 50 min post phage addition was selected for transcriptional analysis, since one step growth experiments revealed a latent period of approximately 90 min for CP_F1 infection. Similarly, no change in the expression of *flaA* was observed in the *flaB* knock-out strain (Supplementary Figures. S5, S6). These data would suggest that the increase in bacteriophage sensitivity or phage yields of *flaB* mutants are not linked to a compensatory increase in *flaA* transcription.

### Disruption of *flaB* affects bacteriophage adsorption

As changes in susceptibility toward phage infection and burst size were observed in the mutant strain, we were interested to examine if a change in adsorption might also be an effect of *flaB* inactivation. The calculated adsorption constant of CP_F1 binding to the PT14 wild type was in a similar range to that of the Cp8unalikevirus phages CP30 and CP8 (Siringan et al., 2014). However, there were 2-fold and greater increases in the adsorption constants (*k*) determined for the *flaB* mutant clones after 30 min incubation (Table 3). Differences in the adsorption constant between the wild type and the mutant were statistically significant for five independent clones based on triplicate experiments (*p* < 0.01), verifying that the absence of FlaB has an effect on the adsorption process.

**Table 3:** **Adsorption constants of phage CP_F1 infecting ***C. jejuni*** PT14 wild type (WT), five independent clones of ***flaB*** mutants and trans-complemented ***flaB*** (***n*** = 3)**.

	***k*-values (× 10^−10^ ml/ min)**	**SD**	***p*-different to WT**
WT	0.54	±0.08	–
*flaB* c1	2.30	±0.16	*p* < 0.01
*flaB* c2	1.31	±0.14	*p* < 0.01
*flaB* c3	3.05	±0.09	*p* < 0.01
*flaB* c4	1.40	±0.15	*p* < 0.01
*flaB* c5	0.97	±0.12	*p* < 0.01
*flaB,0046::flaB*	0.58	±0.06	–

## Discussion

Several reports have demonstrated that *Campylobacter* bacteriophages are dependent on certain surface structures for the infection of suitable hosts, for example capsular polysaccharide or a functional flagella (Coward et al., 2006; Scott et al., 2007; Sørensen et al., 2011; Baldvinsson et al., 2014). These are targets for bacteriophage adsorption, which have been recognized as being prone to variation in expression at different stages of bacterial cell growth and are often subject to stochastic phase variation. Campylobacters contain homopolymer GC tracts within dispensable reading frames that can expand or contract to place the reading frame in or out of phase and the gene effectively on or off (Bayliss et al., 2012; Anjum et al., 2016). Variable expression of surface-associated structures may contribute an obstacle to efficient infection by bacteriophages and may affect the efficacy of phage based biocontrol approaches (Nuijten et al., 1995; Park et al., 2000; Karlyshev et al., 2002; Connerton et al., 2011; Holst Sørensen et al., 2012). In search of phage target structures that do not exhibit phase variability we tested the effect of several genes, recognized as essential for *Campylobacter* virulence and colonization, on the infection process of a collection of bacteriophages. The aim was to find novel elements that may be essential for, or have an impact on, bacteriophage infection. During our screening we found a factor that interferes with the infection process of certain bacteriophages. Inactivation of the minor flagellin gene (*flaB*) showed a number of effects on different parameters of bacteriophage infection. Collectively, these effects increased the efficiency of phage infection and impaired the resilience of the affected host population toward infection. For the first time, we show that the presence of a gene product, classified as a structural protein, confers defensive properties against bacteriophage infection. Further, this effect was not limited to one genetically classified genus of *Campylobacter* myovirus phages, as two phages CP_F1 and CP220 from two different genera showed similar reactions during infection of a flagellin B mutant strain. CP_F1 is a CP8una-like phage and CP220 is the archetype of the CP220-like phages (Javed et al., 2014). Furthermore, these two phages differ in requirements for effective infection. We found CP220 to be flagellotropic, while CP_F1 strictly relies on capsular polysaccharide (CPS). However, CP_F1 also showed a preference for motile cells as infection of *flaA* and *flaAB* mutant strains yielded reduced EOP values.

We hypothesize that *flaB* is an essential component of an integral defensive mechanism against bacteriophage infection. In our observations, disruption of this gene prevented cells from shifting to resistant, non-motile phenotypes during infection of a bacterial lawn on semi-solid media. This phenomenon may be based on the lysis of a large proportion of cells before mutations or gene regulatory responses can develop that lead to a resistant sub-population. In liquid growth cultures, at cell densities below the phage proliferation threshold, bacteriophage CP_F1 was effectively bacteriostatic for *flaB* deficient cultures, whereas the phage did not impair the growth of cultures with an intact *flaB* gene. At cell concentrations that promoted phage proliferation, host bacterial population crashes were observed after phage addition to wild type or mutant cultures. However, the recovery from acute phage infection and the resumption of exponential growth observed for wild type cultures was severely retarded and the growth yields reduced in the *flaB* mutant cultures compared to wild type. These results clearly show that *flaB* deficient strains have a greater susceptibility to phage infection. Inevitably an increase in susceptibility will increase the selective pressure to escape phage infection and the generation of less susceptible sub-populations. Whilst not evident upon infection on semi-solid medium, regrowth was observed post infection of the *flaB* mutants in liquid culture. Spatial differences between liquid and semi-solid media in terms of diffusion, host mobility and distribution might be some of the key factors contributing to the probability of the cells being able to evade infection and develop resistance (Abedon and Yin, 2009). However, it should also be noted that these conditions represent ideal conditions in terms of nutrient availability, suitable oxygen tension and the absence of competitor organisms that would not exist in intestinal or extra-intestinal environments inhabited by campylobacters.

The increase in phage yields observed for the *flaB* mutant are accompanied by a significant increase in the burst size, which suggests that phage maturation is more efficient in the *flaB* mutant. Trans-complementation of the *flaB* mutation results in a reduction in the burst size from 4.2 virions (±1.2) per cell to 1.8 virions (±0.6) per cell, a value similar to that of wild type. The *flaB* mutant also shows an increase in the adsorption constant, which would theoretically make the mutant more susceptible at lower cell densities. Figure 3E conclusively demonstrates that the *flaB* mutant culture remains susceptible to phage infection at cell densities below the phage proliferation threshold, such that host growth is arrested as compared to the wild type and *flaB* trans-complement cultures. Lysis times are predicted to become shorter in phage exhibiting high adsorption rates (Shao and Wang, 2008), and it is noteworthy that the first significant increase in free phage titers were observed for the *flaB* mutant in Figure 4 at 90 min post-infection. From these observations it is evident that any host bacterium that can evolve a countermeasure against this perfect storm would be at a selective advantage when subject to bacteriophage predation. *Campylobacter* bacteriophages have been proposed for the biocontrol of campylobacters in the food chain (Connerton et al., 2011), and from a biotechnological standpoint the introduction of a *flaB* mutant for *Campylobacter* phage amplification strategies offers the prospect of increasing phage yields from growth cultures, which would render large scale phage production more cost effective.

If the FlaB protein acts either directly or indirectly to reduce the burst size, then the *Campylobacter* population would benefit by limiting the release of free virus and the rate of propagation. Such a mechanism could account for the rather low burst size of *Campylobacter* bacteriophages compared to phages infecting other proteobacteria. The peak expression of the *flaB* σ^54^ promoter was reported to occur mid exponential phase (Alm et al., 1993), which would coincide the ideal conditions for efficient phage replication when cells are metabolically replete and at a density beyond the phage proliferation threshold. Under these circumstances the deployment of FlaB as a countermeasure would confer the maximal impact on the bacterial population's ability to evade and adapt to bacteriophage infection.

*Campylobacter* flagella are highly decorated due to O-linked glycosylation of the flagellin monomer (Guerry, 2007; Ewing et al., 2009). A phage receptor binding protein (RBP) has been reported to specifically interact with flagella decorated with acetamidino-modified pseudaminic acid (Javed et al., 2015). As a consequence, changes in the glycosylation pattern of the flagella may introduce or reduce steric effects on the adsorption process. We have measured 1.8–5.6-fold increases in the adsorption rates of *flaB* mutant clones compared to wild type but how this is manifest is unclear since it has been reported that for *C. coli* VC167 surface exposure of the FlaB protein could not be detected (Guerry et al., 1991).

However, it is also reported that inactivation of *flaB* increases colonization efficiency of *C. jejuni* (Wassenaar et al., 1993). Glycosylation is recognized as an essential colonization factor; therefore it is possible that an altered distribution of glycan molecules may affect a change in colonization efficiency (Howard et al., 2009). There is evidence for diversity in glycan structure and variation in the numbers of residues serving as flagellar glycosylation targets in different *Campylobacter* strains (Thibault et al., 2001; Guerry, 2007), and variations in the occupancy of the glycosylation sites adding to the complexity of surface exposed region of the flagellin proteins (Meinersmann and Hiett, 2000; Ulasi et al., 2015). With respect to flagellin sequence in strain C. *jejuni* PT14, FlaA, and B share 95% identity at the protein level. Differences in amino acid composition can be found predominantly within terminal regions of the protein (26 residues). However, a centrally located region of the protein is predicted to be surface exposed, which contains 4 amino acid differences between FlaA and FlaB proteins of *C. jejuni* PT14, one of which is a serine at position 224 in the FlaB sequence that represents an additional substrate for glycosylation. An analysis of the glycan distributions of FlaA and FlaB may provide further insight as to their potential for interaction with bacteriophage.

Information regarding the function of the minor flagellin is sparse, although several studies have characterized *flaB* as not essential for motility (Guerry et al., 1991; Wassenaar et al., 1993; de Vries et al., 2015). These observations lead to the question as to why a paralogous structural gene has emerged and has been preserved throughout all known *Campylobacter* species. It has been reported that intra-genomic recombination between *flaA* and *flaB* can occur in *Campylobacter* cultures (Wassenaar et al., 1995). Later Meinersmann and Hiett (2000) hypothesized that *flaB* could serve as a driver for antigenic variation, since differences in the amino acid position were found in residues that function as targets for O-linked glycosylation. A similar conclusion was reached by Harrington et al. (1997), who identified intra-genomic recombination between *flaA* and *flaB* in strain VC670, and proposed that the variation enabled adaptation to eukaryotic host species, since functional flagella are essential for colonization and a key target of the host immune system. We assume that recombination events between the flagellin genes will also assist in the escape of phage infection by varying the flagellin structure and the O-linked glycosylation attachment sites, a response mechanism that is likely to have evolved before the immune systems of the animal hosts of *Campylobacter* species. However, analyses of the *flaA* coding sequences in this study provided no evidence for recombination events between the *flaA* and *flaB* genes in the escape mutants of bacteriophage infection. Further, no alterations in the *flaA* open reading frame or its 5′-untranslated region were detected. Instead whole genome analysis of escape mutants revealed a single adenine deletion in the N-terminal region of the *rpoN* gene coding for the σ^54^ factor. In most polar flagellate species σ^54^ serves as an essential part of flagellar expression. This is also the case for the flagella biogenesis of *Campylobacter*. A knock-out mutation in the *rpoN* gene of strain C. *jejuni* 11168 resulted in complete absence of flagella and flagellin expression (Jagannathan et al., 2001). Motility loss is one strategy by which campylobacters evade phage infection (Scott et al., 2007; Baldvinsson et al., 2014). Moreover, *Campylobacter* bacteriophage carrier state cultures also escape phage lysis by undergoing growth phase dependent motility attenuation as a response to phage association (Siringan et al., 2014). Whereas, *flaA* gene transcription is down-regulated in carrier state cultures, which likely accounts for their impaired mobility, *flaB* transcription is up-regulated, which based on the current data could be an adaption to limit phage infection leading to cell lysis (Brathwaite et al., 2015; Hooton et al., 2016). It is recognized that σ^54^ is part of a group of transcriptional regulators termed class 1 genes that demonstrate hierarchical regulation of class 2 genes, which are fundamental for the formation of the flagellar secretory apparatus and code for the structural components of the basal body of the flagellum (Hendrixson and DiRita, 2003; Lertsethtakarn et al., 2011). The absence of σ^54^ therefore has a profound effect on the formation of functional flagella. We further found several phase variable genes in the off state. Amongst them were several genes coding for sugar transferases, which may have an impact on the cell surface polysaccharides exposed and the phage infection process. High rates of phase variation in *Campylobacter* can facilitate the adaptation toward environmental effects (Gaasbeek et al., 2009; Bayliss et al., 2012). For example, phase variable frame shifts in the key flagellar export regulator FlhA has been reported to cause transcriptional repression of *flaA* and *flaB* to vary motility in *C. coli* (Park et al., 2000). Further, nucleotide deletions in *fliW* and *flgD* have also been found in connection with motility loss. This phenomenon was observed upon analysis of second-site mutations in a *flaB* knock-out mutant strain (de Vries et al., 2015). To exclude that secondary mutation in connection with *flaB* disruption were responsible for the observed increase in bacteriophage sensitivity; we tested five independent clones, which all carried the selective marker in the desired position. All five independent clones showed identical phenotypes with respect to phage induced lysis and motility. Further, trans-complementation partially restored the wild type phenotypes with respect to cell lysis in spot test assays and continued growth at low cell densities in phage infected broth cultures. These results confirm that the observed changes were introduced through the inactivation of the *flaB* gene.

In our efforts to understand how host genes affect bacteriophage propagation in *C. jejuni* we have identified a novel role for the minor flagellin FlaB. Although not essential for motility, the absence of FlaB makes *C. jejuni* more susceptible to bacteriophage infection. Here we propose that the maintenance of the *flaB* gene is not only an evolutionary adaptation to drive antigenic diversity in response to immune pressure but an earlier adaptation to evade infection by flagellotropic phage, and is maintained as a general countermeasure against bacteriophage propagation. Given the ubiquitous presence of virulent bacteriophages in the environment, it is perhaps not surprising that this seemingly redundant gene duplication was fixed and remains a landmark feature of many *C. jejuni* and *C. coli* strains.

## Author contributions

LL performed the experiments. LL and IC designed the experiments, analyzed the data and wrote the manuscript.

### Conflict of interest statement

The authors declare that the research was conducted in the absence of any commercial or financial relationships that could be construed as a potential conflict of interest.
