# Supplementary material for: The Minor Flagellin of Campylobacter jejuni (FlaB) Confers Defensive Properties against Bacteriophage Infection
Source: Front Microbiol. 2016 Nov 29;7:1908. doi: 10.3389/fmicb.2016.01908 (PMC5126078; doi:10.3389/fmicb.2016.01908)
Supplement: Supplementary file 1 [file Table1.PDF]

Supplementary Table S1: List of primers used in real time qRT-PCR

Primer name	Sequence
mcp_fw	GTATTACTTCGCTTAGAATCTAACTC
mcp_rv	TTATCAACAATTACATCGTAACGATTAT
flaA_q_fw	CAGCTGAGTCACAAATCCGT
flaA_q_rv	CCATGGCATAAGAGCCACTT
flaB_q_fw	GTTAAAGCAGCAGAATCAACCA
flaB_q_rv	ACTCATAGCATAAGAACCTGACTG
rplA_q_fw	GAGATATTGTGGGTAGCGATG
rplA_q_rv	GAGCAACATCCATAGTCACTG

Supplementary Table S2: Swarming motility of escape mutants of *C. jejuni* PT14 from CP_F1 infection spots (Assessment of motility: motile = 20 – 30 mm diameter growth zone, non-motile = < 5 mm diameter growth zone).

Tested clones (n)	motile	non-motile	reduced motility (less then 50%)
100	22	66	12
