# Supplementary material for: The Minor Flagellin of Campylobacter jejuni (FlaB) Confers Defensive Properties against Bacteriophage Infection
Source: Front Microbiol. 2016 Nov 29;7:1908. doi: 10.3389/fmicb.2016.01908 (PMC5126078; doi:10.3389/fmicb.2016.01908)

Supplementary Figure S1: Lysis areas of bacteriophage Cp220 on bacterial lawns of *C. jejuni* PT14. Similar to CP_F1 infection, CP220 showed opaque lysis upon infection of the wild type. In contrast, clear a lysis zone was found in the *flaB* mutant. CP220 was unable to induce lysis of non-motile mutants (*flaA*: major flagellin, *flaB*: minor flagellin, *maf5*: motility accessory factor, *pflA*: paralyzed flagella protein).

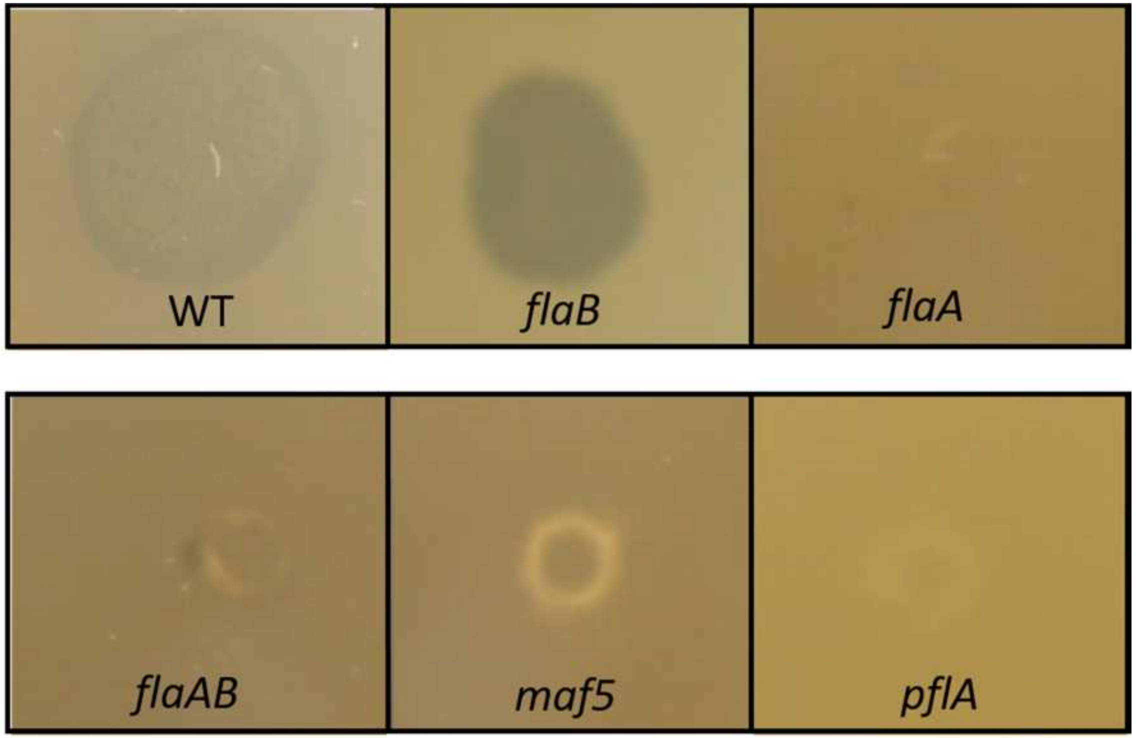

Supplementary Figure S2: A) Swarming motility of five independent clones of *flaB* deficient mutants in *C. jejuni* PT14. The diameters of growth zones after 24 and 48 hours of incubation are derived from 3 biological replicates and presented as means with standard deviations. B) Clear lysis of bacteriophage CP_F1 infecting five independent clones of *flaB* disruption mutants.

A

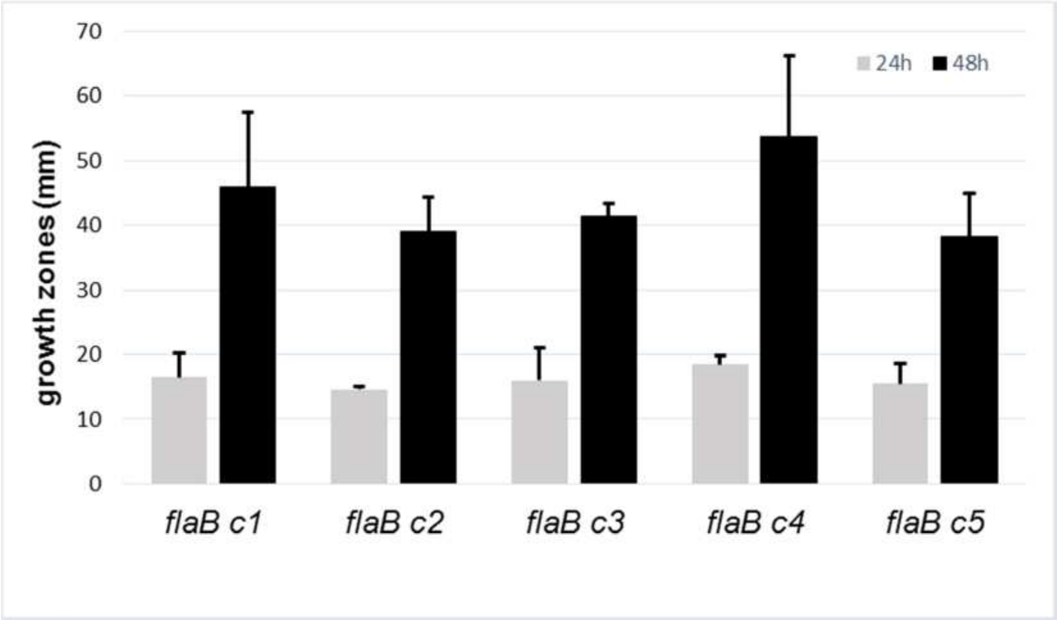

B

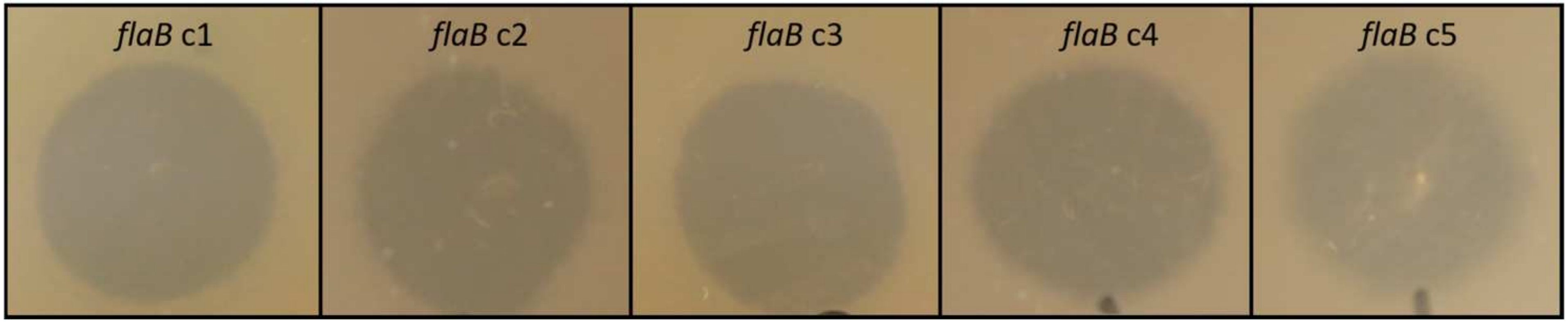

Supplementary Figure S3: Plaque formation of associated bacteriophage CP_F1 in a carrier state culture of PT14 in a growth zone on swarming motility agar (**A**). After collection and serial sub-culturing of cells from the growth zone, phage replication during growth on solid media was observed. The filtered supernatant of an emulsified overnight culture showed lytic activity on a bacterial lawn of a susceptible culture of strain PT14 (**B**).

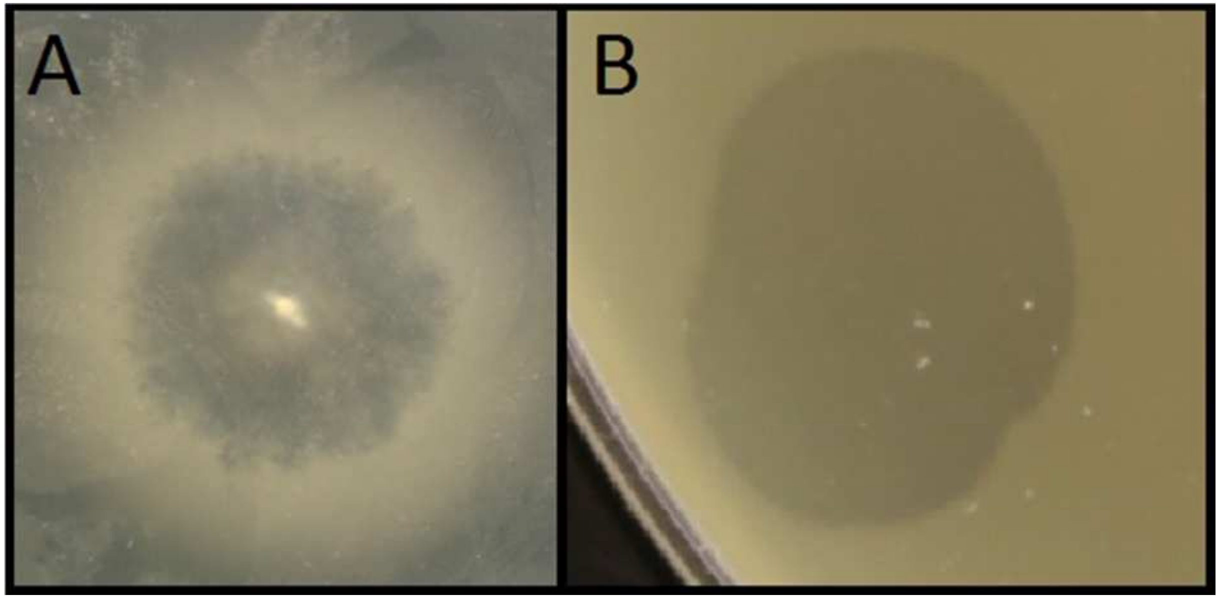

Supplementary Figure S4: Delayed replication of CP_F1 during bacteriophage infection of PT14 wild type and flagellin mutant strain in liquid cultures at initial cell densities below (**A**) and above (**B**) the phage proliferation threshold. During the first 12 hours of infection the phage titres remained static. After 24 hours of incubation an increase of up to 1 $\log_{10}$ of phage release was found in the flagellin mutant, relative to the wild type cultures. Data points represent mean values of n=3 biological replicates with error bars ± standard deviation.

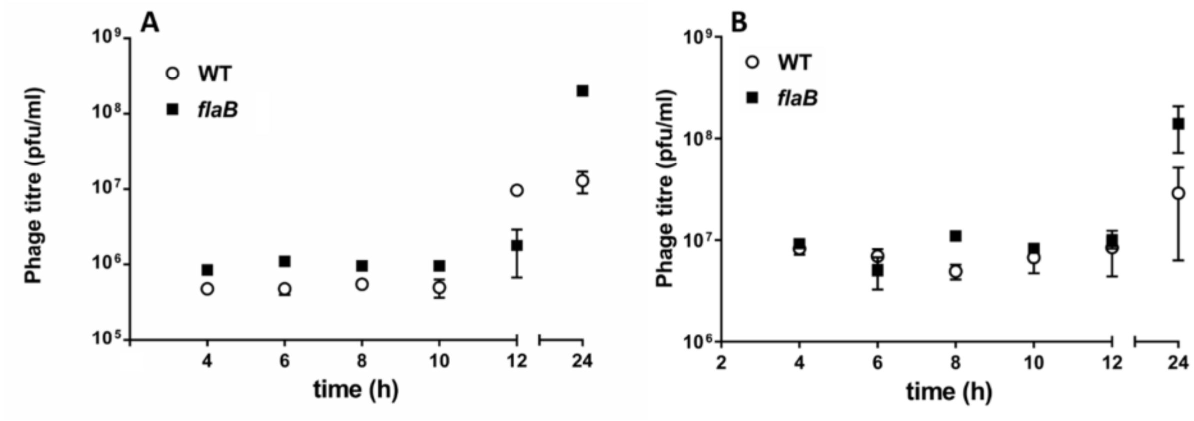

Supplementary Figure S5: Relative expression of flagellin genes of *C. jejuni* PT14 wild type during acute bacteriophage infection (ca. 50 min after phage addition). No significant changes in levels of expressed mRNA were found for either gene relative to the uninfected controls. Samples were normalized against the internal control gene *rplA*. Data represents mean values of three independent experiments carried out with three biological replicates. Error bars show the standard deviation.

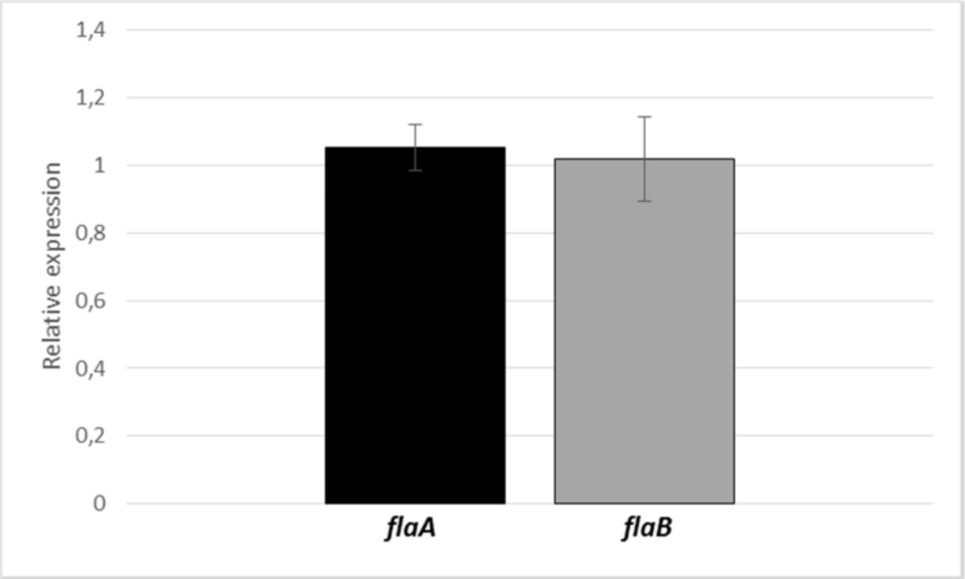

Supplementary Figure S6: Relative expression of the flagellin A gene of *C. jejuni* PT14 *flaB* mutant during acute bacteriophage infection. No significant changes in the levels of expressed mRNA were found relative to the uninfected controls. Samples were normalized against the internal control gene *rplA*. Data represents mean values of three independent experiments carried out with three biological replicates. Error bars show the standard deviation.

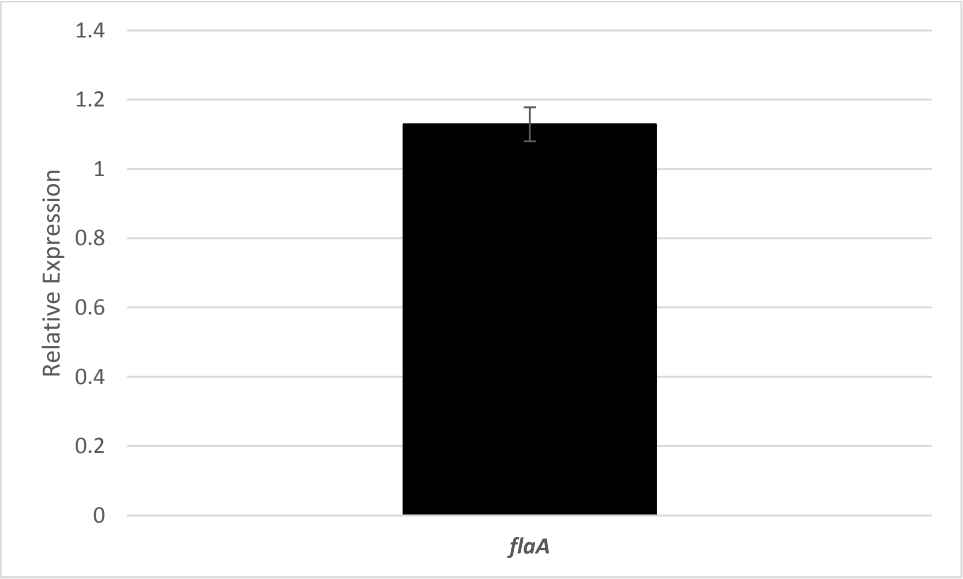

Supplement: Supplementary file 2 [file Image1.PDF]
